# Hábito Alimentar de Idosos Diabéticos e não Diabéticos: Vigitel, Brasil, 2016

**DOI:** 10.36660/abc.20201204

**Published:** 2022-02-14

**Authors:** Daniela de Assumpção, Ana Maria Pita Ruiz, Flavia Silva Arbex Borim, Anita Liberalesso Neri, Deborah Carvalho Malta, Priscila Maria Stolses Bergamo Francisco

**Affiliations:** 1 Universidade Estadual de Campinas Programa de Pós-Graduação em Gerontologia Campinas SP Brasil Universidade Estadual de Campinas (Unicamp) - Programa de Pós-Graduação em Gerontologia,Campinas, SP - Brasil; 2 Universidade de Brasília Departamento de Saúde Coletiva Escola de Ciências da Saúde Brasília DF Brasil Universidade de Brasília (UnB) - Departamento de Saúde Coletiva - Escola de Ciências da Saúde,Brasília, DF - Brasil; 3 Universidade Federal de Minas Gerais Departamento de Enfermagem Materno-Infantil e Saúde Pública Belo Horizonte MG Brasil Universidade Federal de Minas Gerais (UFMG) - Departamento de Enfermagem Materno-Infantil e Saúde Pública,Belo Horizonte, MG - Brasil

**Keywords:** Idoso, Diabetes Mellitus, Ingestão de Alimentos, Inquéritos Epidemiológicos

## Abstract

**Fundamentos:**

A alimentação saudável é um fator de proteção contra o diabetes tipo 2 e desempenha importante papel no tratamento do diabetes e das comorbidades associadas.

**Objetivo:**

Caracterizar o hábito alimentar de idosos diabéticos e não diabéticos com 65 anos ou mais, residentes nas capitais brasileiras e no Distrito Federal.

**Métodos:**

Estudo transversal com dados da pesquisa Vigilância de Fatores de Risco e Proteção para as Doenças Crônicas por Inquérito Telefônico (Vigitel, 2016). Foram estimadas as prevalências de diabetes melito segundo variáveis sociodemográficas, inatividade física, autoavaliação da saúde e índice de massa corporal (IMC). O hábito alimentar foi avaliado pela frequência (semanal e diária) de consumo de alimentos saudáveis e não saudáveis, e pela substituição da comida por lanches. As diferenças foram verificadas por meio do teste Qui-quadrado de Pearson (Rao-Scott) com nível de significância de 5%.

**Resultados:**

Foram entrevistados 13.649 idosos, e a prevalência de diabetes autorreferido foi de 27,2% (IC95%:25,5; 29,0). Nos pacientes diabéticos, observou-se maior consumo de hortaliças cruas (32,1% vs. 26,5%/3-4 dias/semana) e menor de frango (3,8% vs. 6,4%/quase nunca/nunca), suco (24,0% vs. 29,6%) e doces (6,8% vs. 16,2%) ≥5 dias/semana. Os percentuais de idosos com consumo de leite desnatado (51,5% vs. 44,6%) e refrigerante dietético (60,0% vs. 17,3%) ≥5 dias/semana, hortaliças cruas (9,1% vs. 2,5%/no jantar) e doces (37,7% vs. 20,5%/2 vezes/dia) 3-4 dias/semana foram maiores nos diabéticos, comparados aos não diabéticos.

**Conclusão:**

As diferenças observadas sinalizam a necessidade de promover intervenções para alimentação saudável entre todos os idosos, bem como orientações específicas para os diabéticos.

## Introdução

Globalmente, o número de diabéticos passou de 108 milhões (1980) para 422 milhões (2014), representando um aumento de quase 40% ao considerar o crescimento e o envelhecimento populacional.^1^ Em 2019, o Brasil ocupava a 5ª posição (16,8 milhões) entre os 10 países com maior número de portadores de diabetes e a 3ª posição entre os que mais gastavam com o tratamento da doença (52,3 bilhões de dólares).^2^

A Pesquisa Nacional de Saúde de 2013 estimou em 6,2% a prevalência de diabetes na população brasileira (≥18 anos), afetando mais intensamente os indivíduos sem instrução ou com ensino fundamental incompleto, com excesso de peso, hipertensão e colesterol/triglicérides elevados.^3^ Entre os idosos com 65 anos ou mais, 19,8% apresentaram a doença,^3^ indicando o impacto do diabetes nos custos diretos e indiretos para os serviços de saúde, à sociedade e aos portadores da doença.^4^

Uma alimentação baseada em cereais integrais, hortaliças folhosas, frutas, leguminosas, oleaginosas/óleos vegetais ricos em ômega-6, laticínios com baixo teor de gordura e com quantidades restritas de carnes vermelhas/processadas, cereais refinados, doces e bebidas açucaradas desempenha importante papel na prevenção e no manejo do diabetes.^5^ Evidências indicam menor incidência de diabetes com o aumento da ingestão de cereais integrais e farelos de cereais, e maior incidência para o consumo de carne vermelha, carne processada, *bacon* e bebidas açucaradas.^6^ Resultados do Estudo Longitudinal de Saúde do Adulto (ELSA-Brasil 2008-2010 e 2012-2014) mostram que o consumo elevado de carne processada aumentou em 68% a chance de novos casos de resistência insulínica nos homens (>27,1g/dia) e em 23% nas mulheres (> 20,7g/dia); o alto consumo de carne vermelha (>101,9g/dia) elevou em 40% o risco de diabetes em homens.^7^

A análise dos fatores associados ao diabetes para o conjunto de adultos brasileiros (≥18 anos) não revelou diferenças nos hábitos alimentares, considerando o consumo de carne vermelha com gordura e o consumo recomendado de frutas e hortaliças, entre os que relataram ter ou não a doença.^3^ Em uma instituição de referência no tratamento do diabetes que recebe usuários do Sistema Único de Saúde (SUS), observou-se que, entre os padrões alimentares identificados, o “tradicional brasileiro”, caracterizado pelo consumo de arroz, feijão, frango e alimentos regionais, apresentou correlação negativa com os níveis glicêmicos.^8^

Considerando que a alimentação saudável é um fator de proteção contra doenças crônicas não transmissíveis, entre elas o diabetes tipo 2, que desempenha importante papel no tratamento do diabetes e suas comorbidades (p. ex. hipertensão, obesidade e dislipidemia), este estudo teve como objetivo caracterizar o hábito alimentar de idosos diabéticos e não diabéticos com 65 anos ou mais, residentes nas capitais brasileiras e no Distrito Federal.

## Métodos

### Desenho do estudo

Trata-se de estudo transversal de base populacional com entrevistas realizadas por telefone em 2016.

### Contexto

A pesquisa por meio do sistema Vigilância de Fatores de Risco e Proteção para as Doenças Crônicas por Inquérito Telefônico (Vigitel) ocorre anualmente, desde 2006, nas capitais dos estados brasileiros e no Distrito Federal (DF). Apresenta o objetivo de monitorar a frequência e a distribuição dos principais fatores que determinam as doenças crônicas não transmissíveis no Brasil.^9^

### Participantes

No presente estudo, foram incluídos idosos com 65 anos ou mais entrevistados pelo Vigitel 2016. A opção pelo corte etário de 65 anos ou mais foi fundamentada no aumento da expectativa de vida ao nascer, nas mudanças sociodemográficas e políticas observadas no Brasil desde a promulgação do Estatuto do Idoso, em 2003, e na possibilidade de ampliar a comparação dos resultados com os de estudos internacionais.^10^

Os participantes do Vigitel 2016 foram selecionados por meio de amostragem probabilística de adultos (≥18 anos) residentes em domicílios servidos por pelo menos uma linha de telefone fixo em 2016. Em linhas gerais, o plano amostral envolve duas etapas. A primeira consiste no sorteio sistemático e estratificado por código de endereçamento postal (CEP) de pelo menos 5 mil linhas telefônicas em cada capital, a partir do cadastro eletrônico de linhas residenciais fixas das empresas telefônicas. Em seguida, as linhas sorteadas foram ressorteadas e divididas em réplicas de 200 linhas, cada réplica reproduzindo a mesma proporção de linhas por CEP do cadastro original. A segunda etapa do plano amostral consiste no sorteio de um dos adultos residentes no domicílio selecionado, feita após a identificação das linhas residenciais ativas elegíveis.^9^

O Vigitel realiza aproximadamente 2 mil entrevistas em cada cidade, amostra que permite estimar a frequência dos principais fatores de risco para doenças crônicas na população adulta com coeficiente de confiança de 95% e erro máximo de dois pontos percentuais. Cada indivíduo entrevistado recebe um peso pós-estratificação visando à inferência estatística dos resultados para a população adulta de cada capital. Este peso permite igualar a composição sociodemográfica estimada para a população adulta com telefone em cada capital àquela que se estima para a população adulta total da mesma capital, de acordo com as variáveis: sexo, faixa etária e escolaridade.^9^

### Variáveis

As características selecionadas para caracterizar a amostra estudada foram:

- Sociodemográficas: sexo (masculino, feminino); idade (em anos) e escolaridade (0-4, 5-8 e ≥ 9 anos de estudo).
- Inatividade física nos domínios de lazer, trabalho e deslocamento para o trabalho (sim, não).
- Autoavaliação da saúde (muito boa/boa, regular e ruim/muito ruim).
- Índice de massa corporal (IMC = peso [kg]/altura^2^[m]), calculado com informações referidas e classificado em: baixo peso (IMC <22 kg/m^2^), eutrofia (IMC ≥22 e ≤27 kg/m^2^) e excesso de peso (IMC >27 kg/m^2^), conforme o critério da Nutrition Screening Initiative.^11^

### Fonte de dados e mensuração

Foram considerados diabéticos os idosos que responderam sim à questão: “Algum médico já lhe disse que o(a) sr.(a) tem diabetes?” (sim ou não). O hábito alimentar foi avaliado pela frequência de consumo de alimentos saudáveis e não saudáveis, e pela substituição da comida por lanches. O consumo de hortaliças (cruas e cozidas), frutas, suco natural, feijão, leite e frango foi considerado saudável, e o de carnes vermelhas (boi, porco e cabrito), carnes com excesso de gordura (carne vermelha com gordura aparente e frango com pele), leite integral, doces, refrigerantes ou suco artificial e o hábito de substituir as principais refeições (almoço e jantar) por lanches foram considerados não saudáveis. As frequências de consumo dos alimentos foram categorizadas em quase nunca/nunca, 1-2, 3-4 e ≥5 dias na semana. O consumo regular corresponde à frequência ≥5 dias/semana.

### Análise dos dados

Foram estimadas as prevalências de diabetes melito e respectivos intervalos de confiança (IC) de 95%, segundo as variáveis selecionadas para a caracterização dos idosos. Realizaram-se análises estratificadas por escolaridade e idade para verificar a influência dessas características sobre o consumo alimentar. Também foram apresentadas as distribuições de frequências relativas do consumo alimentar para os idosos diabéticos e não diabéticos, assim como a distribuição da frequência semanal de consumo segundo o tipo do alimento, frequência diária de ingestão e hábito de ingerir carne com excesso de gordura. Para verificar as diferenças entre as proporções, foi utilizado o teste Qui-quadrado de Pearson (Rao-Scott) com nível de significância de 5%.

A análise estatística foi realizada com auxílio do programa Stata versão 15.1, considerando o delineamento complexo de amostragem do estudo.

### Considerações éticas

Os objetivos da pesquisa Vigitel foram informados aos participantes na ocasião do contato telefônico e o consentimento livre e esclarecido foi substituído pelo consentimento verbal. O estudo foi aprovado pela Comissão Nacional de Ética em Pesquisa em Seres Humanos, do Ministério da Saúde (Parecer nº 355.590 de 26/06/2013).

## Resultados

**Figura 1:**
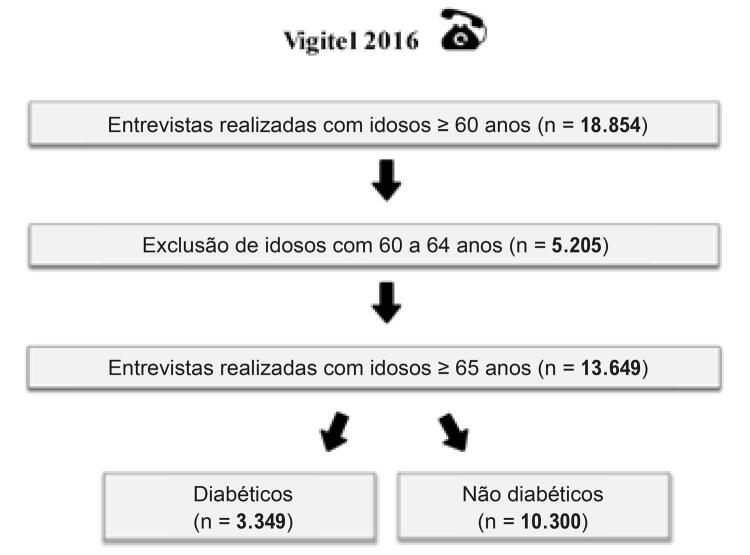
– Processo de composição da amostra. Fonte: Vigitel, Brasil, 2016.

Dos 18.854 idosos entrevistados, 5.205 foram excluídos deste estudo por apresentarem idade menor que 65 anos. Portanto, foram analisadas informações de 13.649 idosos, dos quais 3.349 eram diabéticos e 10.300, não diabéticos ( Figura 1 ). A prevalência de diabetes autorreferido foi 27,2% (IC_95%_: 25,5; 29,0). A média de idade foi 73,6 anos (IC_95%_: 73,2; 74,1) nos idosos diabéticos e de 73,8 anos (IC_95%_: 73,5; 74,0) nos não diabéticos. Na comparação desses subgrupos, os diabéticos apresentaram maior proporção de inatividade física (p=0,015), de pior avaliação subjetiva da saúde e de excesso de peso (p<0,001). Quanto à escolaridade, foi observada menor proporção de diabéticos com 9 anos ou mais de estudo (p<0,001) ( Tabela 1 ).

**Tabela 1:** – Distribuição dos idosos diabéticos (n=3.349) e não diabéticos (n=10.300), segundo características sociodemográficas, inatividade física, autoavaliação da saúde e índice de massa corporal. Vigitel, Brasil, 2016

Variáveis	Diabéticos		Não diabéticos		Valor de p^c^

	n^a^	%^b^ (IC95%)	n^a^	%^b^ (IC95%)	
**Sexo**					
Masculino	1.081	35,2 (31,7;38,9)	3.252	37,7 (35,5;39,9)	0,254
Feminino	2.268	64,8 (61,1;68,3)	7.048	62,3 (60,1;64,4)	
**Escolaridade (em anos de estudo)**					
0-4	1.165	51,0 (47,3;54,8)	3.039	45,9 (43,7;48,1)	**0,015**
5-8	669	24,8 (21,6;28,2)	1.992	24,8 (22,9;26,7)	
≥ 9	1.515	24,2 (21,7;26,9)	5.269	29,3 (27,7;30,9)	
**Inatividade física nos três domínios** ^**d**^					
Não	2.046	55,4 (51,6;59,2)	7.125	67,0 (65,0;69,0)	**< 0,001**
Sim	1.303	44,6 (40,8;48,4)	3.175	32,9 (31,0;35,0)	
**Autoavaliação da saúde**					
Muito boa/boa	1.300	40,1 (36,4;43,9)	6.148	59,9 (57,8;62,0)	**< 0,001**
Regular	1.629	47,6 (43,8;51,4)	3.505	34,2 (32,2;36,2)	
Ruim/muito ruim	380	12,3 (10,1;15,0)	537	5,9 (4,9;7,0)	
**Categorias de IMC** ^**e**^					
Baixo peso	257	8,0 (6,3;10,2)	1.351	15,6 (14,1;17,3)	**< 0,001**
Eutrofia	1.113	39,3 (35,3;43,5)	4.226	48,0 (45,7;50,4)	
Excesso de peso	1.532	52,6 (48,5;56,7)	3.339	36,3 (34,2;38,6)	

*^a^ n: número de indivíduos na amostra não ponderada. ^b^ %: percentual ponderado para ajustar a distribuição sociodemográfica da amostra Vigitel à distribuição da população adulta de cada cidade projetada para 2016; IC95%: intervalo de confiança de 95%. ^c^ Valor de p do teste Qui-quadrado de Pearson. ^d^ Domínios de atividade física: lazer, trabalho e deslocamento para o trabalho. ^e^ IMC: índice de massa corporal. Fonte: Vigitel, Brasil, 2016.*

Os resultados da análise da frequência do consumo regular de alimentos estratificada segundo níveis de escolaridade (0-8 e ≥9 anos) em idosos diabéticos e não diabéticos, são mostrados na Tabela 2 . Os resultados indicam menor consumo regular de leite desnatado/semidesnatado e maior de hortaliças cruas no almoço e no jantar entre os diabéticos com baixa escolaridade. Entre os não diabéticos, observou-se maior proporção de idosos com até 8 anos de estudo ingerindo leite integral, carne vermelha com gordura e hortaliças cruas no jantar em 5 ou mais dias/semana (ver Tabela 2 ).

**Tabela 2:** – Frequência do consumo regular (≥5 dias/semana) de alimentos segundo níveis de escolaridade (em anos), em idosos diabéticos e não diabéticos. Vigitel, Brasil, 2016.

Variáveis	Diabéticos		p^a^	Não diabéticos		p^a^
	
	Escolaridade (em anos)			Escolaridade (em anos)		
	
	0-8	9 ou +		0-8	9 ou +	
**Tipo de leite**			**0,009**			**<0,001**
Integral	45,7	34,8		**53,1**	**40,2**	
Desnatado/semidesnatado	**48,9**	**60,7**		**40,8**	**53,7**	
Ambos	5,5	4,5		6,1	6,1	
**Como costuma comer carne vermelha**			0,813			**0,008**
Retira a gordura	71,6	71,2		**66,6**	**77,8**	
Come com a gordura	22,8	25,0		**30,1**	**19,1**	
Não come carne com muita gordura	5,6	3,8		3,3	3,1	
**Como costuma comer frango**			0,155			0,084
Retira a pele	88,9	78,3		83,1	90,4	
Come com a pele	10,6	19,5		13,0	5,6	
Não come frango com pele	0,5	2,2		3,9	4,0	
**Tipo de refrigerante**			0,544			0,089
Normal	34,9	35,5		75,4	63,5	
*Diet/light/zero*	61,6	55,7		13,8	25,5	
Ambos	3,6	8,8		10,8	11,0	
**Hortaliças cruas**			**0,010**			**0,002**
Almoço	63,1	73,7		70,0	74,3	
Jantar	4,0	10,6		**5,9**	**1,4**	
Ambos	**32,9**	**15,7**		24,1	24,3	
**Hortaliças cozidas**			0,487			0,893
Almoço	53,4	60,0		63,6	64,0	
Jantar	8,1	4,5		4,4	5,0	
Ambos	38,5	35,5		32,0	30,9	
**Frutas**			0,322			0,369
1 vez por dia	37,4	37,8		41,2	38,5	
2 vezes por dia	39,7	34,5		38,4	38,6	
≥3 vezes por dia	22,9	27,7		20,3	22,9	
**Suco natural**			0,143			0,191
1 copo	57,2	46,5		53,1	52,2	
2 copos	24,1	34,0		27,8	32,3	
≥3 copos	18,7	19,4		19,1	15,4	
**Refrigerante**			0,453			0,817
1 a 2 copos/latas por dia	67,9	76,8		80,0	81,2	
≥3 copos/latas por dia	32,1	23,2		20,0	18,8	
**Doces**			0,097			0,971
1 vez ao dia	44,9	60,5		64,6	63,8	
2 vezes ao dia	22,0	28,7		23,7	24,7	
≥3 vezes ao dia	33,1	10,8		11,7	11,5	

*^a^ Valor de p do teste Qui-quadrado de Pearson (Rao-Scott). Valores em negrito indicam diferenças estatisticamente significativas. Fonte: Vigitel, Brasil, 2016.*

Observaram-se diferenças estatisticamente significativas na frequência de consumo de hortaliças cruas, frango, suco natural e doces entre idosos diabéticos e não diabéticos ( Tabela 3 ). Os percentuais de diabéticos que nunca/quase nunca ingeriam carne de frango foi inferior ao verificado entre os não diabéticos. A ingestão de leite (1-2 dias/semana) foi menor nos diabéticos. O consumo de suco natural e doces (≥ 5 dias/semana) foi menor entre os idosos que apresentavam a doença. A maioria dos diabéticos não ingeria doces, no entanto, quase 15% incluíam esses alimentos no repertório alimentar (≥3 dias/semana). Não houve diferenças em relação à substituição das grandes refeições por lanches, mas cerca de 20% dos diabéticos comiam lanches no jantar.

**Tabela 3:** – Distribuição da frequência semanal de consumo de alimentos saudáveis e não saudáveis e de outros hábitos alimentares em idosos diabéticos e não diabéticos (n=13.649). Vigitel, Brasil, 2016.

Variáveis de consumo alimentar	Diabéticos				Não diabéticos				Valor de p^b^

	QN/N^a^	1-2	3-4	≥ 5	QN/N^a^	1-2	3-4	≥ 5	

	Dias na semana				Dias na semana				
Feijão	4,6	15,3	17,7	62,4	4,6	16,0	19,9	59,5	0,496
Hortaliças cruas	7,7	22,2	**32,1**	38,0	8,9	25,5	**26,5**	39,1	**0,029**
Hortaliças cozidas	4,2	32,1	35,4	28,3	4,9	33,0	33,1	29,0	0,665
Frutas	2,5	11,4	17,5	68,6	3,8	12,5	19,1	64,6	0,144
Leite	17,8	**6,6**	7,4	68,2	16,7	**9,8**	6,6	66,9	0,094
Carne vermelha	8,9	40,9	31,7	18,5	11,8	36,3	33,0	18,9	0,062
Frango	**3,8**	41,2	41,4	13,6	**6,4**	40,5	38,2	14,9	**0,034**
Suco natural	31,1	26,6	18,3	**24,0**	28,4	24,1	17,9	**29,6**	**0,039**
Refrigerante	54,7	27,9	7,1	10,3	52,8	29,0	8,4	9,8	0,632
Doces	**53,9**	31,2	**8,1**	**6,8**	**33,3**	36,6	**13,9**	**16,2**	**< 0,001**
Substituição do almoço por lanches	83,5	13,8	2,4	0,3	85,7	12,0	1,8	0,5	0,365
Substituição do jantar por lanches	45,4	23,9	10,2	20,5	45,4	21,3	8,6	24,7	0,088

*^**a**^ Quase nunca ou nunca. ^**b**^ Valor de p do teste Qui-quadrado de Pearson; valores em negrito indicam diferenças estatisticamente significativas. Fonte: Vigitel, Brasil, 2016.*

A Tabela 4 mostra os resultados da frequência de consumo semanal dos idosos diabéticos e não diabéticos de acordo com as características dos alimentos e a frequência diária de consumo. Os diabéticos apresentaram menores percentuais de ingestão de leite integral e maiores de leite desnatado, comparados aos não diabéticos. O consumo de refrigerante dietético foi mais elevado entre os diabéticos. Considerando a frequência semanal de 3 a 4 vezes, observou-se maior percentual de consumo de hortaliças cruas (jantar) e doces (2 vezes por dia) nos idosos com diabetes.

**Tabela 4:** – Distribuição da frequência semanal de consumo de alimentos em idosos diabéticos e não diabéticos segundo o tipo, a frequência diária de ingestão e o costume de ingerir carne com excesso de gordura. Vigitel, Brasil, 2016.

Variáveis	Diabéticos			Não diabéticos			p^a^1-2	p^a^3-4	p^a^≥ 5

	1-2	3-4	≥ 5	1-2	3-4	≥ 5			

	Dias na semana			Dias na semana					
**Tipo de leite**							**0,002**	0,839	**0,035**
Integral	47,2	62,8	43,3	68,7	60,6	49,3			
Desnatado/semidesnatado	48,5	33,8	51,5	28,4	35,1	44,6			
Ambos	4,3	3,4	5,2	2,9	4,3	6,1			
**Como costuma comer carne vermelha**							0,826	0,844	0,515
Retira a gordura	84,4	82,2	71,5	83,6	80,5	70,0			
Come com a gordura	10,4	13,4	23,3	11,7	14,8	26,7			
Não come carne com muita gordura	5,2	4,4	5,2	4,7	4,7	3,3			
**Como costuma comer frango**							0,191	0,516	0,194
Retira a pele	83,8	85,8	86,3	80,9	88,4	84,9			
Come com a pele	15,5	12,3	12,8	17,3	9,8	11,2			
Não come frango com pele	0,7	1,9	0,9	1,8	1,8	3,9			
**Tipo de refrigerante**							**<0,001**	**0,001**	**<0,001**
Normal	39,4	39,4	35,0	71,0	70,4	71,9			
*Diet* / *light* /zero	50,1	49,1	60,0	21,2	23,6	17,3			
Ambos	10,5	11,5	5,0	7,8	6,0	10,8			
**Hortaliças cruas**							0,312	**0,002**	0,334
Almoço	83,0	75,3	66,4	83,1	81,5	71,7			
Jantar	2,7	9,1	6,0	5,4	2,5	4,1			
Ambos	14,3	15,6	27,6	11,5	16,0	24,2			
**Hortaliças cozidas**							0,144	0,868	0,134
Almoço	67,6	67,5	55,3	73,2	65,4	63,8			
Jantar	9,0	8,1	7,1	9,7	8,4	4,7			
Ambos	23,4	24,4	37,6	17,1	26,2	31,5			
**Frutas**							0,566	0,627	0,359
1 vez por dia	69,0	54,6	37,5	65,3	52,1	40,3			
2 vezes por dia	23,0	31,3	38,3	28,3	30,0	38,5			
≥3 vezes por dia	8,0	14,1	24,2	6,4	17,9	21,2			
**Suco natural**							0,989	0,354	0,758
1 copo	63,9	60,2	54,2	64,3	55,6	52,8			
2 copos	27,6	29,8	27,0	27,4	30,8	29,4			
≥3 copos	8,5	10,0	18,8	8,3	13,6	17,8			
**Refrigerante**							0,134	0,597	0,144
1 a 2 copos/latas por dia	97,4	80,4	70,4	95,0	84,5	80,4			
≥3 copos/latas por dia	2,6	19,6	29,6	5,0	15,5	19,6			
**Doces**							0,355	**0,020**	0,086
1 vez ao dia	80,1	57,3	51,9	78,2	72,1	64,3			
2 vezes ao dia	17,7	37,7	25,0	17,4	20,5	24,1			
≥3 vezes ao dia	2,2	5,0	23,1	4,4	7,4	11,6			

*^a^ p: Valor de p do teste Qui-quadrado de Pearson da comparação entre diabéticos e não diabéticos; em negrito, p < 0,05. Fonte: Vigitel, Brasil, 2016.*

## Discussão

Este estudo buscou caracterizar o hábito alimentar de idosos diabéticos e não diabéticos, residentes nas capitais brasileiras e no Distrito Federal e, dentre os resultados, observou-se que idosos diabéticos com baixa escolaridade apresentaram proporção inferior de consumo regular de leite com teor reduzido de gordura e proporção superior de hortaliças cruas (almoço e jantar).

Em Pelotas/RS (2010), um estudo transversal verificou que 67,6% dos idosos ingeriam leite integral e 32,4%, desnatado ou semidesnatado; para o conjunto da população analisada, foram identificadas menores prevalências de consumo de leite desnatado ou semidesnatado nos segmentos com baixos níveis de escolaridade (<12 anos de estudo).^12^ A concentração de gordura dos tipos de leite fluído fica em torno de 3,7% para o integral, entre 0,6% e 2,9% para o semidesnatado, e atinge, no máximo, 0,5% para o desnatado. Embora seja recomendado o tipo desnatado para diabéticos,^4^ na elaboração do plano alimentar, é necessário respeitar a preferência e a situação de vida dos indivíduos, considerando que, de modo geral, não apresenta boa aceitação entre os idosos e que a remoção da gordura diminui o teor energia e vitaminas lipossolúveis.^13 , 14^

Em famílias com baixa renda e escolaridade, a estrutura do cardápio cotidiano é composta por arroz, feijão e uma “mistura”, como carne ou vegetais, no almoço e no jantar,^15^ o que poderia explicar, parcialmente, o maior consumo regular de hortaliças cruas entre os diabéticos com baixa escolaridade. Em idosos do município de Campinas/SP (2008-2009), a qualidade da dieta, avaliada pela ingestão de 12 componentes alimentares, foi significativamente maior nos mais velhos (≥ 80 anos *vs.* 60-69 anos) e nos diabéticos, que pode decorrer da preocupação com a saúde, levando o indivíduo a adotar as recomendações dos profissionais da saúde para uma alimentação mais saudável.^16^ O consumo alimentar saudável é determinado pela posição socioeconômica, como demonstrado por um estudo com dados da Pesquisa Nacional de Saúde, no qual pessoas com níveis mais elevados de escolaridade e renda apresentaram maiores prevalências de consumo de hortaliças, frutas, suco de frutas, peixes, leite desnatado/semidesnatado e carnes sem gordura aparente.^17^

A frequência de consumo de hortaliças cruas, frango, leite, suco natural e doces diferiu entre diabéticos e não diabéticos. Também foram observadas diferenças no tipo de leite e refrigerante ingerido e na frequência diária de consumo de hortaliças cruas e doces. Mudanças no estilo de vida, incluindo a adoção de hábitos alimentares saudáveis, são fundamentais para a prevenção, o tratamento do diabetes tipo 2 e para a redução do risco de complicações decorrentes da doença.^2 , 4 , 18^

A proporção de diabéticos que nunca/quase nunca comiam frango foi menor do que a de não diabéticos. Uma metanálise de três coortes realizadas entre 1986-2006, 1980-2008 e 1991-2005 com adultos norte-americanos revelou que a substituição diária de uma porção de 85g de carne vermelha e processada por frango ou peixe reduzia em 10% o risco de diabetes tipo 2.^19^ A associação entre o consumo de carne vermelha e processada e a incidência de diabetes tem sido explicada por vários fatores, entre eles, o excesso de ferro no organismo humano, o aumento do estresse oxidativo, o teor de gordura saturada e a presença de sódio e conservantes nas carnes processadas.^19^

Foi observada menor proporção de consumo de leite de 1 a 2 vezes por semana nos idosos com diabetes e maior proporção de consumo de leite desnatado ou semidesnatado. Uma revisão sistemática de 53 estudos realizados de 2007-2018 não encontrou associação significativa para o risco de diabetes e o consumo de leite com alto (risco relativo [RR] = 0,99; IC_95%_: 0,88-1,11) e baixo teor de gordura (RR = 1,01; IC_95%_: 0,98-1,05), considerando um incremento de 200g/dia, assim como para iogurte (incremento de 50g/dia, RR = 0,94; IC_95%_: 0,91-0,98) e laticínios (200g/dia, RR = 0,96; IC_95%_: 0,94-0,99).^6^ Uma pesquisa analisou dados de três coortes prospectivas com profissionais da saúde (1986-2012, 1984-2012, 1991-2013) e não observou associação entre consumo de laticínios com gordura e risco de diabetes, comparado com as calorias de carboidratos; também foi verificado que a substituição equivalente de 5% das calorias de gorduras lácteas por outras gorduras de origem animal aumentava 17% o risco de diabetes.^20^

Os idosos com diabetes apresentaram proporções mais elevadas de consumo de hortaliças cruas (3 a 4 vezes/semana). Uma metanálise de estudos prospectivos publicados de 1997 a 2014 identificou que maior ingestão de hortaliças folhosas reduziu o risco de diabetes.^5^ Um estudo de coorte europeu (1993-2004) com 3.704 participantes e com informações de sete diários alimentares detectou associação inversa para incidência de diabetes e maior quantidade (mediana = 2,6 porções/dia de 80g) e variedade (média = 11,4 itens/semana) de hortaliças.^21^ Habitualmente, as hortaliças são ingeridas junto com arroz, feijão e outros alimentos que compõem o almoço e o jantar, o que pode acarretar no menor consumo de alimentos de alta densidade energética e baixa densidade nutricional; uma alimentação rica em hortaliças, especialmente as folhosas, fornece uma variedade de compostos bioativos, que atuam na prevenção da doença.^21^ O achado destaca a importância de orientações para promover o consumo regular de hortaliças no almoço e no jantar para o conjunto dos idosos. Deve-se considerar que problemas de saúde bucal (p. ex., cárie dentária, doença periodontal e edentulismo) podem dificultar a mastigação de hortaliças mais fibrosas, por isso é necessário empregar técnicas de cocção adequadas à textura de cada tipo de hortaliça.

Foi observado que quase 46% dos diabéticos ingeriam dois ou mais copos de suco por dia, regularmente. Um estudo com dados de coortes prospectivas norte-americanas (1984-2008, 1991-2009, 1986-2008) detectou que o consumo de suco de frutas (≥ 1 porção/dia) aumentava em 21% o risco de desenvolver diabetes; observou-se, também, que a substituição do suco por frutas inteiras reduzia em 7% o risco da doença, especialmente mirtilo (-33%), uva/passas (-19%), ameixa seca (-18%), maçã/pera (-14%) e banana (-13%).^22^ Fatores como a carga glicêmica, o conteúdo de fibras alimentares e outros nutrientes e o estado líquido podem explicar a associação entre ingestão de sucos de frutas e ocorrência de diabetes.^22^ O preparo do suco pode prejudicar o conteúdo de fibra alimentar e outros nutrientes, além de favorecer a rápida absorção da glicose.^23^

Não foi verificada associação estatística entre o costume de ingerir carne vermelha com gordura entre idosos com e sem diabetes, mas cerca de um quarto dos diabéticos ingeriam regularmente carne com excesso de gordura. As carnes vermelhas apresentam quantidades importantes de ácidos graxos (AG) saturados e colesterol, e, ainda, as carnes processadas têm alto teor de sódio e conservantes, como nitratos e nitritos, estando associadas ao maior risco de diabetes.^5 , 18 , 19^ Uma metanálise com seis estudos prospectivos evidenciou que o consumo diário de 100g de carnes vermelhas e 50g de carnes processadas aumentava em 19% e 51% a incidência de diabetes, respectivamente.^19^

A qualidade do carboidrato e da gordura é mais importante do que a quantidade ingerida, destacando o papel dos carboidratos de baixo índice glicêmico/carga glicêmica, dos AG ômega-6 e das dietas predominantemente de origem vegetal para a prevenção e controle do diabetes.^5 , 18^ Em relação ao perfil lipídico das dietas, ressalta-se a importância do equilíbrio no consumo de AG ômega-6/ômega-3.^24 , 25^ A alimentação da sociedade contemporânea é caracterizada pelo consumo excessivo de AG ômega-6, encontrados principalmente em óleos vegetais ricos em ácido linoleico (p. ex.: milho, soja, girassol), comumente empregados na fabricação de alimentos processados e ultraprocessados, e pelo consumo insuficiente de AG ômega-3, presentes em peixes, óleos de peixes, sementes/óleos de chia, linhaça e hortaliças verde-escuras, ricos em ácido α-linolênico.^24 - 26^ O excesso de AG ômega-6 na dieta provoca um estado inflamatório, que está relacionado com o desenvolvimento de várias doenças crônicas, incluindo as cardiovasculares.^24 , 25^

Neste estudo, o consumo de doces (2 vezes por dia) e refrigerantes dietéticos foi maior nos diabéticos. Os doces/sobremesas apresentam elevado teor de energia e carboidratos simples de adição, e são dispensáveis em uma alimentação saudável. No entanto, são alimentos que fazem parte da cultura alimentar e não são proibidos aos diabéticos, mas é necessário que o consumo seja controlado em termos de frequência e porcionamento.^4^ A ingestão de refrigerantes deve ser evitada, independentemente do tipo. Um estudo de coorte multicêntrico com mulheres de 50 a 79 anos constatou que a maior frequência de consumo de refrigerante *diet* (≥ 2 vezes/dia) elevava o risco de acidente vascular cerebral e mortalidade.^27^ Resultados de outros estudos revelam que a ingestão de refrigerantes aumenta o risco de doença hepática gordurosa não alcoólica por mecanismos relacionados ao metabolismo da frutose.^28 , 29^ De modo geral, o plano alimentar para diabéticos deve atender as recomendações energéticas e nutricionais definidas para o estágio de vida, e basear-se em alimentos *in natura* e minimamente processados, tais como leguminosas, cereais, hortaliças e frutas, evitando alimentos de baixa qualidade nutricional como os ultraprocessados.^4 , 5^ Ressalta-se ainda orientação de alternar os óleos vegetais usados no preparo dos alimentos e fazer misturas com azeite de oliva para obter proporções mais adequadas de AG linoleico e α-linolênico.

Os percentuais de idosos diabéticos que ingeriam bebidas adoçadas e doces foram elevados. Os açúcares de adição presentes nas bebidas adoçadas (refrigerantes/refrescos) e nos doces induzem a resistência à insulina e a hiperinsulinemia, representando um fator de risco para o desenvolvimento de doenças cardiovasculares e diabetes tipo 2.^18 , 22 , 30^ Portanto, a recomendação de evitar ou limitar o consumo de carnes vermelhas/processadas e açúcares de adição se justifica pelos benefícios no controle dos fatores de risco cardiometabólicos relacionados ao diabetes, como excesso de peso, hipertensão arterial e dislipidemia.^4 , 18 , 19^

A adesão dos diabéticos às orientações nutricionais depende, em parte, do respeito às condições socioeconômicas, culturais, familiares e às preferências alimentares do indivíduo. Um estudo qualitativo com diabéticos tipo 2, sem complicações crônicas da doença, não medicados com insulina, atendidos por serviços de atenção básica em um município do interior paulista, mostrou que a prescrição nutricional é reconhecida como essencial no controle do diabetes, mas que o significado de controle da dieta é peculiar, resultante de ajustes nos modos de comer que considerem os gostos e a vida social.^31^ Em ocasiões de comensalidade, o consumo de alimentos e bebidas não recomendados por profissionais da saúde pode ser uma opção do indivíduo para não afetar a sociabilidade.^31 , 32^

Os resultados deste estudo mostram que ainda são grandes os desafios para promover e viabilizar o acesso à alimentação saudável para os idosos em geral. A avaliação do Plano de Ações Estratégicas para o Enfrentamento das Doenças Crônicas não Transmissíveis no Brasil identificou avanços nas metas de redução do consumo regular de refrigerantes e aumento do consumo de frutas e hortaliças,^33^ atualização do Guia Alimentar para a População Brasileira e aprovação das novas regras de rotulagem nutricional.^26 , 33 , 34^ Apesar dos importantes avanços na área da alimentação, ainda não foram implantadas medidas regulatórias pelo Estado, como a taxação de alimentos e bebidas ultraprocessados, e incentivo fiscal para estimular a produção, a comercialização e o consumo de frutas e hortaliças.

Entre as limitações, destaca-se o viés de seleção da amostra, composta por indivíduos que possuíam linha telefônica residencial fixa. No entanto, o uso de fatores de ponderação minimiza diferenças observadas nas populações com e sem telefone e o peso de pós-estratificação permite que as estimativas sejam extrapoladas para a totalidade dos indivíduos (com e sem telefone).^9^ Outra limitação refere-se ao possível viés de recordatório especialmente quanto à frequência de consumo dos alimentos. O desenho transversal do inquérito Vigitel impossibilita conhecer as relações de temporalidade das associações observadas entre diabetes e consumo alimentar, ou seja, não é possível avaliar se o diagnóstico da doença e as orientações recebidas produziram mudanças na alimentação, como verificado em relação ao consumo de refrigerante *diet* . Os inquéritos telefônicos são pesquisas rápidas, que produzem dados confiáveis e de baixo custo para o monitoramento da prevalência de fatores de risco e condições de saúde das populações.

## Conclusão

Os resultados deste estudo indicam diferenças no hábito alimentar de idosos diabéticos e não diabéticos com relação a hortaliças cruas, leite, frango, suco natural, refrigerantes e doces. Ressalta-se a necessidade de promover a alimentação adequada e saudável nessa população, com orientações realizadas por nutricionista no âmbito da atenção básica com equipe multiprofissional, e que sejam adequadas à situação de vida dos idosos.
