# Supplementary material for: Hábito Alimentar de Idosos Diabéticos e não Diabéticos: Vigitel, Brasil, 2016
Source: Arq Bras Cardiol. 2022 Feb 14;118(2):388–97. [Article in Portuguese] doi: 10.36660/abc.20201204 (PMC8856680; doi:10.36660/abc.20201204)
Supplement: Translation

# Translation: Eating Behavior of Older Adults with and Without Diabetes: The Vigitel Survey, Brazil, 2016

**Authors:** Daniela de Assumpção, Ana Maria Pita Ruiz, Flavia Silva Arbex Borim, Anita Liberalesso Neri, Deborah Carvalho Malta, Priscila Maria Stolses Bergamo Francisco

**Affiliations:** 1 Brazil Universidade Estadual de Campinas (Unicamp) - Programa de Pós-Graduação em Gerontologia, Campinas, SP - Brazil; 2 Brazil Universidade de Brasília (UnB) - Departamento de Saúde Coletiva - Escola de Ciências da Saúde, Brasília, DF - Brazil; 3 Brazil Universidade Federal de Minas Gerais (UFMG) - Departamento de Enfermagem Materno-Infantil e Saúde Pública, Belo Horizonte, MG - Brazil

**Keywords:** Aged, Diabetes Mellitus, Eating, Health Surveys

## Abstract

**Background:**

A healthy diet is a protection factor against type 2 diabetes and plays an important role in the treatment of the disease, as well as associated comorbidities.

**Objective:**

Characterize the eating habits of older adults (≥ 65 years) with and without diabetes residing in capital cities and the Federal District of Brazil.

**Methods:**

A cross-sectional study was conducted using data from the Surveillance of Risk and Protection Factors for Chronic Diseases Through a Telephone Survey (Vigitel, 2016). The prevalence of diabetes mellitus was estimated according to sociodemographic variables, physical inactivity level, self-rated health status and body mass index. Dietary habits were assessed based on the frequency (weekly and daily) of consumption of healthy and unhealthy foods and the replacement of food by snacks. Differences were determined using Pearson’s chi-square test (Rao-Scott), with the significance level set at 5%.

**Results:**

A total of 13,649 older adults were interviewed. The prevalence of self-reported diabetes was 27.2% (95% CI: 25.5; 29.0). Compared to non-diabetics, diabetic individuals had a higher consumption of raw vegetables (32.1% vs. 26.5%/3-4 days/week) and lower consumption of chicken (3.8% vs. 6.4%/hardly ever/never), fruit juice (24.0% vs. 29.6%) and sweets (6.8% vs. 16.2%) ≥ 5 days/week. Compared to non-diabetics, diabetic individuals consumed more skim milk (51.5% vs. 44.6%) and diet soda (60.0% vs. 17.3%) ≥ 5 days/week, raw vegetables (9.1% vs. 2.5%/at dinner) and sweets (37.7% vs. 20.5%/twice/day) 3-4 days/week.

**Conclusion:**

The observed differences emphasize the need for healthy eating interventions for all older adults, as well as specific counseling for those with diabetes.

## Introduction

On the global scale, the number of individuals with diabetes increased from 108 million in 1980 to 422 million in 2014. Taking into account the increase and aging of the population, this represents an increase of nearly 40%.^1^ In 2019, Brazil occupied the 5^th^position (16.8 million) among the 10 countries with the highest number of diabetic individuals and 3^rd^position among those that spent most on the treatment of the disease (52.3 billion US dollars).^2^

The 2013 National Health Survey estimated the prevalence of diabetes in the adult population (≥ 18 years) in Brazil to be 6.2%, with the disease exerting a greater effect on individuals with no schooling or an incomplete elementary school education, as well as those with excess weight, hypertension and high cholesterol/triglyceride levels.^3^ The prevalence among older adults (≥ 65 years) was 19.8%,^3^ which indicates the impact of the disease on direct and indirect costs for healthcare services, society and affected invididuals.^4^

A diet based on whole grains, leafy vegetables, fruits, legumes, seeds/vegetable oils rich in omega-6 and dairy products with a low fat content, as well as restricted quantities of red/processed meats, refined grains, sweets and sweetened beverages plays an important role in the prevention and management of diabetes.^5^ Evidence indicates a lower incidence of diabetes with the increase in the consumption of whole grains and bran and a greater incidence with the increase in the consumption of red/processed meats, bacon and sweetened beverages.^6^ Results from the 2008-2010 and 2012-2014 Brazilian Longitudinal Study of Adult Health] [ *Estudo Longitudinal de Saúde do Adulto* (ELSA)] show that the high consumption of processed meats increased the likelihood of new cases of insulin resistance by 68% in men (> 27.1 g/day) and 23% in women (> 20.7 g/day) and the high consumption of red meat (> 101.9 g/day) increased the risk of diabetes by 40% in men.^7^

An analysis of factors associated with diabetes in Brazilian adults (≥ 18 years) revealed no differences in dietary habits (consumption of fatty red meats and recommended intake of fruits and vegetables) between those who reported having or not having the disease.^3^ At a reference institution for the treatment of diabetes that received individuals from the public healthcare system, the “traditional Brazilian” diet, which is characterized by the consumption of rice, beans, chicken and regional foods, was negatively correlated with blood sugar levels.^8^

As healthy eating is a protection factor against chronic noncommunicable diseases, including type 2 diabetes, and plays an important role in the treatment of diabetes, as well as associated comorbidities (e.g., hypertension, obesity and dyslipidemia), the aim of the present study was to characterize the eating habits of older adults (≥ 65 years) with and without diabetes residing in capital cities and the Federal District of Brazil.

## Methods

### Study design

A population-based cross-sectional study was conducted using data from telephone interviews held in 2016.

### Context

The Surveillance of Risk and Protection Factors for Chronic Diseases Through a Telephone Survey (Vigitel) has occurred annually since 2006 in state capitals and the Federal District of Brazil. The aim of the Vigitel survey is to monitor the frequency and distribution of the main factors that determine chronic noncommunicable diseases in the country.^9^

### Participants

The present study involved older adults (≥ 65 years) interviewed during the 2016 Vigitel survey. The choice of this age group was based on the increase in life expectancy, sociodemographic and political changes in Brazil since the decree of the Older Adult Statute in 2003 and the possibility of comparing the results with those of international studies.^10^

The participants in the 2016 Vigitel survey were selected through probabilistic sampling of adults (≥ 18 years) residing in households with at least one residential telephone line in 2016. The two-stage sampling was performed. The first stage consisted of a systematic drawing (stratified by postal code) of at least five thousand telephone lines in each capital city using the electronic records of landlines of telephone companies. The selected lines were submitted to another drawing and divided into replicates of 200 lines, with each replicate reproducing the same proportion of lines per postal code of the original records. The second stage consisted of the drawing of one of the adults residing in the selected household after the identification of the eligible active residential lines.^9^

Vigitel conducts approximately two thousand interviews in each city, which enables estimating the frequency of the main risk factors for chronic diseases in the adult population with a 95% confidence interval and maximum error of two percentage points. Each interviewee receives a post-stratification weight to enable the statistical inference of the results for the adult population in each city. Weighting ensures the linking of the sociodemographic composition estimated for the adult population with a telephone line in each capital city to that estimated for the total adult population of the same city in terms of gender, age group and level of schooling.^9^

### Variables

The following characteristics were selected to characterize the sample:

- Sociodemographic: gender (male, female); age (in years); level of schooling (0-4, 5-8 and ≥ 9 years of study);
- Physical inactivity level at leisure and work and when commuting to work (yes, no);
- Self-rated health status (very good/good, fair and poor/very poor);
- Body mass index (BMI = weight [kg]/height [m]^2^ ), calculated from thr reported information and classified as underweight (< 22 kg/m^2^ ), ideal weight range (22 to ≤ 27 kg/m^2^ ) and excess weight (BMI > 27 kg/m^2^ ) based on the criteria of the Nutrition Screening Initiative.^11^

### Data source and measurement

Older adults who answered yes to the following question were considered to be diabetic: “ *Has any physician ever told you that you have diabetes?* ” (yes or no). Dietary habits were assessed based on the frequency of the consumption of healthy and unhealthy foods, as well as the replacement of food for by snacks. The consumption of vegetables (raw and cooked), fruits, natural fruit juice, beans, milk and chicken was considered healthy. The consumption of red meat (beef, pork and goat), fatty meats (red meat with visible fat and chicken with skin), whole milk, sweets, sweetened beverages (soda and artificial juice) and the habit of replacing a main meal (lunch or dinner) by snacks were considered unhealthy. Food intake frequencies were categorized as hardly ever/never, 1-2, 3-4 and ≥ 5 days per week. Regular consumption corresponded to ≥ 5 days/week.

### Data analysis

The prevalence of *Diabetes mellitus* was estimated with its respective 95% confidence intervals (CI) according to the variables selected for sample characterization. The analyses were stratified by level of schooling and age to determine the influence of these characteristics on dietary habits. The distribution of relative frequencies of food intake, as well as of weekly consumption according to the type of food, daily intake and the habit of consuming fatty meats were presented for older adults with and without diabetes. The differences between proportions were determined using Pearson’s chi-square test (Rao-Scott) with the significance level set at 5% (p > 0.05). The statistical analyses were performed with the aid of the Stata program, version 15.1, considering the complex sampling design.

### Ethical considerations

The participants received clarifications regarding the objectives of the Vigitel survey during telephone contact. Written consent was substituted by verbal consent. This study received approval from the National Human Research Ethics Committee of the Health Ministry (certificate number355.590, of June 26, 2013).

## Results

Among of 18,854 older adults (≥ 60 years of age) interviewed, 5,205 were excluded from the present study for being younger than 65 years of age. Thus, data from 13,649 older adults were analyzed, 3,349 of whom were diabetic and 10,300 were not diabetic ( Figure 1 ). The prevalence of self-reported diabetes was 27.2% (95%CI : 25.5 to 29.0). Mean age was 73.6 years (95%CI: 73.2 to 74.1) among those with diabetes and 73.8 years (95%CI: 73.5 to 74.0) among those without diabetes. Compared to non-diabetics, individuals with diabetes had greater proportions of physical inactivity (p = 0.015), worse self-rated health status and excess weight (p < 0.001). The diabetics also had a lower proportion of schooling with nine or more years of study (p < 0.001) ( Table 1 ).

**Figure 1:** – Sample composition process. Vigitel, Brazil, 2016.

**Table 1:** – Distribution of diabetic (n = 3349) and non-diabetic (n = 10,300) older adults according to sociodemographic characteristics, physical inactivity, self-rated health status and body mass index. Vigitel, Brazil, 2016

Variables	Diabetics		Non-diabetics		p-value^c^

	n^a^	%^b^(95%CI)	n^a^	%^b^(95%CI)	
Gender					
Male	1081	35.2 (31.7;38.9)	3252	37.7 (35.5;39.9)	0.254
Female	2268	64.8 (61.1;68.3)	7048	62.3 (60.1;64.4)	
Level of schooling (years of study)					
0-4	1165	51.0 (47.3;54.8)	3039	45.9 (43.7;48.1)	**0.015**
5-8	669	24.8 (21.6;28.2)	1992	24.8 (22.9;26.7)	
≥ 9	1515	24.2 (21.7;26.9)	5269	29.3 (27.7;30.9)	
Physical inactivity in 3 domains^d^					
No	2046	55.4 (51.6;59.2)	7125	67.0 (65.0;69.0)	**< 0.001**
Yes	1303	44.6 (40.8;48.4)	3175	32.9 (31.0;35.0)	
Self-rated health status					
Very good/good	1300	40.1 (36.4;43.9)	6148	59.9 (57.8;62.0)	**< 0.001**
Fair	1629	47.6 (43.8;51.4)	3505	34.2 (32.2;36.2)	
Poor/very poor	380	12.3 (10.1;15.0)	537	5.9 (4.9;7.0)	
BMI categories^e^					
Underweight	257	8.0 (6.3;10.2)	1351	15.6 (14.1;17.3)	**< 0.001**
Ideal weight range	1113	39.3 (35.3;43.5)	4226	48.0 (45.7;50.4)	
Excess weight	1532	52.6 (48.5;56.7)	3339	36.3 (34.2;38.6)	

*a) n: number of individuals in the unweighted sample; b) %: percentage weighted to adjust sociodemographic distribution of Vigitel sample to distribution of the adult population in each city projected for 2016; CI: confidence interval; c) p-value of Pearson’s chi-square test; d) Physical activity domains: leisure, work and commuting to work; e) BMI: body mass index.*

The results of the analysis of regular food intake stratified by schooling (0-8 and ≥ 9 years) in diabetic and non-diabetic older adults are displayed in Table 2 . Lower regular consumption of reduced-fat/skim milk and greater consumption of raw vegetables at lunch and dinner were found among diabetics with low level of schooling. Among non-diabetics, a higher proportion of those with up to eight years of schooling consumed whole milk, fatty red meat and raw vegetables at dinner five or more days/week ( Table 2 ).

**Table 2:** – Frequency of regular consumption (≥ 5 days/week) of foods according to the level of schooling (in years) in diabetic and non-diabetic older adults. Vigitel, Brazil, 2016

Variables	Diabetics		p^a^	Non-Diabetics		p^a^
	
	Level of schooling (years)			Level of schooling (years)		
	
	0-8	9 or +		0-8	9 or +	
**Type of milk**			**0.009**			**<0.001**
Whole	45.7	34.8		**53.1**	**40.2**	
Skim/reduced-fat	**48.9**	**60.7**		**40.8**	**53.7**	
Both	5.5	4.5		6.1	6.1	
**How do you usually eat red meat**			0.813			**0.008**
Removes fat	71.6	71.2		**66.6**	**77.8**	
Eats with fat	22.8	25.0		**30.1**	**19.1**	
Does not eat meat with much fat	5.6	3.8		3.3	3.1	
**How do you usually eat chicken**			0.155			0.084
Removes skin	88.9	78.3		83.1	90.4	
Eats with skin	10.6	19.5		13.0	5.6	
Does not eat chicken with skin	0.5	2.2		3.9	4.0	
**Type of soda**			0.544			0.089
Normal	34.9	35.5		75.4	63.5	
Diet/light/zero	61.6	55.7		13.8	25.5	
Both	3.6	8.8		10.8	11.0	
**Raw vegetables**			**0.010**			**0.002**
Lunch	63.1	73.7		70.0	74.3	
Dinner	4.0	10.6		**5.9**	**1.4**	
Both	**32.9**	**15.7**		24.1	24.3	
**Cooked vegetables**			0.487			0.893
Lunch	53.4	60.0		63.6	64.0	
Dinner	8.1	4.5		4.4	5.0	
Both	38.5	35.5		32.0	30.9	
**Fruits**			0.322			0.369
Once a day	37.4	37.8		41.2	38.5	
Twice a day	39.7	34.5		38.4	38.6	
≥ 3 times a day	22.9	27.7		20.3	22.9	
**Natural fruit juice**			0.143			0.191
1 glass	57.2	46.5		53.1	52.2	
2 glasses	24.1	34.0		27.8	32.3	
≥ 3 glasses	18.7	19.4		19.1	15.4	
**Soda**			0.453			0.817
1 to 2 glasses/cans a day	67.9	76.8		80.0	81.2	
≥ 3 glasses/cans a day	32.1	23.2		20.0	18.8	
**Sweets**			0.097			0.971
Once a day	44.9	60.5		64.6	63.8	
Twice a day	22.0	28.7		23.7	24.7	
≥ 3 times a day	33.1	10.8		11.7	11.5	

*^a^p-value of Pearson’s chi-square test (Rao-Scott). Values in bold type indicate statistically significant differences.*

Significant differences between diabetics and non-diabetics were found for the intake frequency of raw vegetables, chicken, natural fruit juice and sweets ( Table 3 ). The percentage of diabetics that hardly ever/never consumed chicken was lower than that found in the non-diabetics. Milk intake (1-2 days/week) was lower among diabetics. The consumption of natural fruit juice and sweets (≥ 5 days/week) was lower among diabetics. The majority of diabetics did not consume sweets, but nearly 15.0% included these foods in their diet (≥ 3 days/week). No significant difference was found regarding the replacement of meals by snacks, but approximately 20.0% of the diabetics ate snacks for dinner.

**Table 3:** – Distribution of the weekly frequency of healthy and unhealthy food consumption and other dietary habits in diabetic and non-diabetic older people. Vigitel, Brazil, 2016 (n = 13,649)

Food intake variables	Diabetics				Non-Diabetics				p-value^b^

	HE/N^a^	1-2	3-4	≥ 5	HE/N^a^	1-2	3-4	≥ 5	

	Days per week				Days per week				
Beans	4.6	15.3	17.7	62.4	4.6	16.0	19.9	59.5	0.496
Raw vegetables	7.7	22.2	**32.1**	38.0	8.9	25.5	**26.5**	39.1	**0.029**
Cooked vegetables	4.2	32.1	35.4	28.3	4.9	33.0	33.1	29.0	0.665
Fruits	2.5	11.4	17.5	68.6	3.8	12.5	19.1	64.6	0.144
Milk	17.8	**6.6**	7.4	68.2	16.7	**9.8**	6.6	66.9	0.094
Red meat	8.9	40.9	31.7	18.5	11.8	36.3	33.0	18.9	0.062
Chicken	**3.8**	41.2	41.4	13.6	**6.4**	40.5	38.2	14.9	**0.034**
Natural fruit juice	31.1	26.6	18.3	**24.0**	28.4	24.1	17.9	**29.6**	**0.039**
Soda	54.7	27.9	7.1	10.3	52.8	29.0	8.4	9.8	0.632
Sweets	**53.9**	31.2	**8.1**	**6.8**	**33.3**	36.6	**13.9**	**16.2**	**< 0.001**
Replacement of lunch by snacks	83.5	13.8	2.4	0.3	85.7	12.0	1.8	0.5	0.365
Replacement of dinner by snacks	45.4	23.9	10.2	20.5	45.4	21.3	8.6	24.7	0.088

*^**a**^ hardly ever or never;^**b**^ p-value of Pearson’s chi-square test; Values in bold type indicate statistically significant differences.*

Table 4 displays the weekly intake frequencies in the diabetic and non-diabetic groups according to the characteristics of the foods and daily intake frequency. Diabetics had lower percentages of the consumption of whole milk and higher percentages of skim milk consumption compared to non-diabetics. The consumption of diet soda was higher among the diabetics. Considering a weekly frequency of 3-4 times, greater intakes of raw vegetables (dinner) and sweets (twice per day) were found in the diabetics.

**Table 4:** – Distribution of the weekly frequency of food intake in diabetic and non-diabetic older adults according to type, daily intake frequency and habit of eating meat with excess fat. Vigitel, Brazil, 2016

Variables	Diabetics			Non-Diabetics			p^a^1-2	p^a^3-4	p^a^≥ 5

	1-2	3-4	≥ 5	1-2	3-4	≥ 5			

	Days per week			Days per week					
Type of milk							**0.002**	0.839	**0.035**
Whole	47.2	62.8	43.3	68.7	60.6	49.3			
Skim/reduced-fat	48.5	33.8	51.5	28.4	35.1	44.6			
Both	4.3	3.4	5.2	2.9	4.3	6.1			
How do you usually eat red meat							0.826	0.844	0.515
Removes fat	84.4	82.2	71.5	83.6	80.5	70.0			
Eats with fat	10.4	13.4	23.3	11.7	14.8	26.7			
Does not eat meat with much fat	5.2	4.4	5.2	4.7	4.7	3.3			
How do you usually eat chicken							0.191	0.516	0.194
Removes skin	83.8	85.8	86.3	80.9	88.4	84.9			
Eats with skin	15.5	12.3	12.8	17.3	9.8	11.2			
Does not eat chicken with skin	0.7	1.9	0.9	1.8	1.8	3.9			
Type of soda							**<0.001**	**0.001**	**<0.001**
Normal	39.4	39.4	35.0	71.0	70.4	71.9			
Diet/light/zero	50.1	49.1	60.0	21.2	23.6	17.3			
Both	10.5	11.5	5.0	7.8	6.0	10.8			
Raw vegetables							0.312	**0.002**	0.334
Lunch	83.0	75.3	66.4	83.1	81.5	71.7			
Dinner	2.7	9.1	6.0	5.4	2.5	4.1			
Both	14.3	15.6	27.6	11.5	16.0	24.2			
Cooked vegetables							0.144	0.868	0.134
Lunch	67.6	67.5	55.3	73.2	65.4	63.8			
Dinner	9.0	8.1	7.1	9.7	8.4	4.7			
Both	23.4	24.4	37.6	17.1	26.2	31.5			
Fruits							0.566	0.627	0.359
Once a day	69.0	54.6	37.5	65.3	52.1	40.3			
Twice a day	23.0	31.3	38.3	28.3	30.0	38.5			
≥ 3 times a day	8.0	14.1	24.2	6.4	17.9	21.2			
Natural fruit juice							0.989	0.354	0.758
1 glass	63.9	60.2	54.2	64.3	55.6	52.8			
2 glasses	27.6	29.8	27.0	27.4	30.8	29.4			
≥ 3 glasses	8.5	10.0	18.8	8.3	13.6	17.8			
Soda							0.134	0.597	0.144
1 to 2 glasses/cans a day	97.4	80.4	70.4	95.0	84.5	80.4			
≥ 3 glasses/cans a day	2.6	19.6	29.6	5.0	15.5	19.6			
Sweets							0.355	**0.020**	0.086
Once a day	80.1	57.3	51.9	78.2	72.1	64.3			
Twice a day	17.7	37.7	25.0	17.4	20.5	24.1			
≥ 3 times a day	2.2	5.0	23.1	4.4	7.4	11.6			

*^a^p-value of Pearson’s chi-square test of the comparison between diabetics and non-diabetics; significant differences (p < 005) in bold type.*

## Discussion

The present study sought to characterize the eating habits of older adults with and without diabetes residing in state capitals and the Federal District of Brazil. Among the findings, diabetic individuals with low level of schooling had a lower proportion of the regular consumption of reduced-fat/skim milk and a higher proportion of the regular consumption of raw vegetables at lunch and dinner.

A cross-sectional study conducted in 2010 in the city of Pelotas (southern Brazil) found that 67.6% of older adults consumed whole milk and 32.4% consumed skim or reduced-fat milk. In the overall analyzed population, lower frequencies of skim or reduced-fat milk intake were found aming individuals with lower levels of schooling (< 12 years of study).^12^ The concentration of fat is around 3.7% in whole milk, between 0.6% and 2.9% in reduced-fat milk and a maximum of 0.5% in skim milk. Although skim milk is recommended for diabetics,^4^ the design of a dietary plan should respect the preferences and living situation of individuals, considering that skim milk is generally not well accepted by older adults and the removal of the fat reduces the energy content and quantity of liposoluble vitamins.^13 , 14^

In families with low income and level of schooling, the daily menu consists of rice, beans and a source of animal protein, such as meat, or vegetables at lunch and dinner,^15^ which may partially explain the greater consumption of raw vegetables among diabetics with a low level of schooling. In a survey conducted in 2008-2009 in the city of Campinas (southeastern Brazil), diet quality (assessed by the consumption of 12 dietary components) in older adults was significantly better among the oldest individuals (≥ 80 years *versus* 60-69 years) and diabetics, which may stem from health concerns, leading individuals to adopt the recommendations of healthcare providers for a healthier diet.^16^ Healthy eating is determined by one’s socioeconomic position, as demonstrated in a study using data from the National Health Survey, in which individuals with higher levels of schooling and income had higher frequencies of the consumption of vegetables, fruits, fruit juices, fish, reduced-fat/skim milk and meat without visible fat.^17^

The frequency of the consumption of raw vegetables, chicken, natural fruit juice and sweets differed between the diabetics and non-diabetics. Moreover, differences were found regarding the type of milk and soda consumed, as well as the daily intake of raw vegetables and sweets. Changes in lifestyle, including the adoption of healthy eating habits, are fundamental in the prevention and treatment of type 2 diabetes, as well as the reduction in the risk of disease complications.^2 , 4 , 18^

The proportion of diabetics who hardly ever/never consumed chicken was lower compared to that found in the non-diabetics. A meta-analysis of three cohort studies conducted in 1986-2006, 1980-2008 and 1991-2005 involving American adults revealed that the daily replacement of a 85-g portion of red meat and processed meat by chicken or fish reduced the risk of type 2 diabetes by 10%.^19^ The association between the consumption of red and processed meats and the incidence of diabetes has been explained by several factors, including the excess of iron in the organism, the increase in oxidative stress, the saturated fat content and the presence of sodium and preservatives in processed meats.^19^

A proportion of milk intake lower than 1-2 times a week and a higher proportion of the consumption of skim or reduced-fat milk were found in the individuals with diabetes. A systematic review of 53 studies conducted between 2007 and 2018 found no significant association between the risk of diabetes and the consumption of milk with high fat (relative risk [RR] = 0.99; 95%CI: 0.88 to 1.11) or low fat content (RR = 1.01; 95%CI: 0.98 to 1.05), considering an increase of 200 g/day; the same was true for yogurt (increase of 50 g/day, RR = 0.94; 95%CI: 0.91 to 0.98) and other dairy products (200 g/day, RR = 0.96; 95%CI: 0.94 to 0.99).^6^ A study analyzing data from three prospective cohorts involving healthcare providers (1986-2012, 1984-2012 and 1991-2013) found no association between the consumption of dairy products with fat and the risk of diabetes compared to calories from carbohydrates; moreover, the replacement of 5% of calories from dairy fat by fat from an animal source increased the risk of diabetes by 17%.^20^

The older adults with diabetes had higher proportions of the consumption of raw vegetables (3-4 times/week). A meta-analysis of prospective studies published between 1997 and 2014 found that a greater intake of leafy vegetables reduced the risk of diabetes.^5^ A European cohort study (1993-2004) involving 3,704 participants and information on seven foods consumed daily detected an inverse association between the incidence of diabetes and a greater quantity (median = 2.6 portions/day of 80 g) and variety (mean = 11.4 items/week) of vegetables.^21^ Vegetables are normally consumed with rice, beans and other foods that constitute lunch and dinner, which may lead to the lower consumption of foods with high energy and low nutritional density. A diet rich in vegetables, especially leafy greens, provides a variety of bioactive compounds that contribute to the disease prevention.^21^ This finding underscores the importance of counseling for the promotion of the regular consumption of vegetables at lunch and dinner by all older people. However, one should bear in mind that oral health problems (e.g., dental caries, periodontal disease and edentulism) can hinder the chewing of more fibrous vegetables. It is therefore necessary to employ cooking techniques that are adequate for the texture of each type of vegetable.

Nearly 46% of the diabetics regularly consumed two or more glasses of juice per day. A study with data from American cohorts (1984-2008, 1991-2009 and 1986-2008) found that the consumption of fruit juices (≥ 1 portion/day) increased the risk of developing diabetes by 21% and also found that the replacement of juice by the whole fruit reduced the risk of the disease by 7%, especially blueberries (-33%), grapes/raisins (-19%), prunes (-18%), apples/pears (-14%) and bananas (-13%).^22^ Factors, such as the glycemic load, fiber and nutrient content and the liquid state may explain the association between fruit juices and the occurrence of diabetes.^22^ Moreover, preparing juice lowers the content of fiber and other nutrients and favors the fast absorption of glucose.^23^

No significant difference was found regarding the habit of eating fatty red meat between the individuals with and without diabetes, but approximately one-quarter of the diabetics regularly consumed meat with excess fat. Red meat has important quantities of saturated fatty acids and cholesterol and processed meats have a high content of sodium and preservatives, such as nitrates and nitrites, which are associated with a greater risk of diabetes.^5 , 18 , 19^ A meta-analysis with six prospective studies revealed that the daily intake of 100 g of red meat and 50 g of processed meat increased the incidence of diabetes by 19% and 51%, respectively.^19^

The quality of carbohydrates and fat is more important than the consumed amount, as carbohydrates with a low glycemic index/glycemic load, omega-6 fatty acids and predominantly plant-based diets play an important role in the prevention and control of diabetes.^5 , 18^ Moreover, it is important to have the adequate balance of omega-6 and omega-3 fatty acids.^24 , 25^ The diet of contemporary society is characterized by excessive intake of omega-6 fatty acids, which are mainly found in vegetable oils rich in linoleic acid (e.g., corn, soybean and sunflower seed oil) commonly employed in the manufacturing of processed and ultraprocessed foods, and insufficient omega-3 intake, which is found in fish, fish oils, chia and flax seedoils, and dark green vegetables rich in α-linolenic acid.^24 - 26^ The excess of omega-6 fatty acids in the diet provokes an inflammatory state, which is related to the development of several chronic diseases, including cardiovascular disease.^24 , 25^

In the present study, the consumption of sweets (twice a day) and diet soda was greater in the diabetics. Sweets/desserts have a high energy content and added simple carbohydrates, which are dispensable in a healthy diet. However, these foods are part of the eating culture and are not prohibited for diabetics, but it is necessary to control consumption in terms of frequency and portion.^4^ The consumption of soda should be avoided, regardless of the type. A multicenter cohort study with women 50 to 79 years of age found that the greater frequency of diet soda intake (≥ twice/day) raised the risk of stroke and mortality.^27^ Results from other studies showed that the consumption of soda increases the risk of non-alcoholic fatty liver disease through mechanisms related to the metabolism of fructose.^28 , 29^ In general, the dietary plan for diabetics should meet the energy and nutritional recommendations for the stage of life and should be based on whole foods and minimally processed foods, such as legumes, grains, fruits and vegetables, with the avoidance of foods of low nutritional quality, such as ultra-processed products.^4 , 5^ Moreover, one should alternate vegetable oils used in the preparation of foods and make blends with olive oil to obtain more adequate proportions of linoleic and α-linolenic acids.

The percentages of diabetic older adults who consumed sweetened beverages and sweets were high. The added sugar in sugar-sweetened soft drinks and sweets induces insulin resistance and hyperinsulinemia and is a risk factor for the development of cardiovascular disease and type 2 diabetes.^18 , 22 , 30^ Therefore, the recommendation to avoid or limit the consumption of red/processed meats and added sugar is justified by the benefits in the control of cardiometabolic risk factors related to diabetes, such as excess weight, arterial hypertension and dyslipidemia.^4 , 18 , 19^

The diabetics’ adherence f to nutritional counseling partially depends on socioeconomic, cultural and familial characteristics, as well as individual food preferences. A qualitative study involving individuals with type 2 diabetes (without chronic complications of the disease and not taking insulin) in the primary care setting in a city in the state of São Paulo, Brazil, found that the nutritional prescription is recognized as essential for diabetes control, but the meaning of diet control is unique, resulting from adjustments in eating habits that consider one’s tastes and social life.^31^ At gatherings, an individual may choose to consume foods and beverages not recommended by healthcare providers as to not affect sociability.^31 , 32^

The results of this study emphasize the considerable challenges in promoting and ensuring access to a healthy diet for older adults in general. The assessment of the Strategic Action Plan for Combating Chronic Noncommunicable Diseases in Brazil has recognized advances in goals related to a reduction in the regular consumption of soda and an increase in the consumption of fruits and vegetables,^33^ as well as the updating of the Dietary Guide for the Brazilian Population and the approval of new nutritional labeling rules.^26 , 33 , 34^ Despite important advances in the field of diet and nutrition, no regulatory measures have been implemented by the government, such as taxation on ultraprocessed foods and beverages and fiscal incentives to stimulate the production, sales and consumption of fruits and vegetables.

The present study has limitations that should be considered. One limitation regards selection bias, as the sample comprised individuals with a residential telephone line only. However, the use of weighting factors minimizes differences in populations with and without a telephone line and post-stratification weighting enables the estimates to be extrapolated to the totality of individuals (with and without a residential telephone line).^9^ Another limitation regards a possible recall bias, especially regarding the frequency of food consumption. The cross-sectional design of the Vigitel survey prevents the determination of temporality relations in the associations found between diabetes and food intake, meaning that it is not possible to determine whether the diagnosis of the disease and counseling resulted in changes in eating habits, as found in relation to the consumption of diet soda. Telephone surveys are quick and produce reliable data at low cost for the monitoring the prevalence of risk factors and health conditions in populations.

## Conclusion

The results of the present study indicate differences in the eating habits of older adults with and without diabetes in terms of the consumption of raw vegetables, milk, chicken, natural fruit juice, soda and sweets. It is necessary to encourage adequate, healthy eating habits in this population, with counseling provided by nutritionists from the multidisciplinary teams in primary care that is adequate to the living conditions of older people.
